# Book Reviews

**Published:** 1918-05

**Authors:** 


### BOOK REVIEWS

**Books mentioned may be inspected at and ordered through this office. So far as possible, books received in any month will be reviewed in the issue of the second month following. Pamphlets, quarterly and similar periodicals, reports, transactions, etc., will, as a rule, merely be mentioned.**

**A Text-Book of the Practice of Medicine,** by James M. Anders, M. D., Ph. D., LL.D., Professor of Medicine and Clinical Medicine, Medico-Chirurgical College Graduate School, University of Pennsylvania, Thirteenth Edition Thoroughly Revised with the Assistance of John H. Musser, Jr., M. D., Associate in Medicine, University of Pennsylvania. Octavo of 1259 pages, fully illustrated. Philadelphia and London: W. B. Saunders Company, 1917. Cloth, $6.00 net; Half Morocco, $7.50 net.

The twelfth edition of this book was published in 1915. Since then it has been extensively revised and entirely reset, making literally a new volume which preserves all those features that have made it so popular for the last twenty years. We note that the part on the disease of the nervous system—about 200 pages—has been rewritten; also several sections dealing with prophylactic vaccination, specific therapy, splenic anemia, the arrhythmias, intestinal toxemia, bacteriology of pertussis, hemolytic jaundice and pellagra. Among additions to the former text may be noted particularly acidosis, treatment of asthma, estimations of hepatic and renal function, anaphylaxis of food intoxication, focal sepsis, pneumococcic infections and pyorrhea alveolaris. In diagnosis, much emphasis is placed on differential features, many of which are brought into contrast in the 70 or more diagnostic tables. In presenting the treatment of disease due attention is always given to the author's main idea, which is "casual treatment." This book will continue to hold a high place among the working tools of the practitioner.

---

**Diseases of the Chest and the Principles of Physical Diagnosis,** by George W. Norris, M. D., Assistant Professor of Medicine in the University of Pennsylvania, and Henry R. M. Landis, M. D., Assistant Professor of Medicine in the University of Pennsylvania, with a chapter on the Electrocardiograph in Heart Disease, by Edward B. Krumbharr, Ph. D., M. D., Assistant Professor of Research Medicine in the University of Pennsylvania. Octavo volume of 782 pages with 413 illustrations. Philadelphia and London: W. B. Saunders Company, 1017. Cloth, $7.00 net. Half Morocco, $8.50 net.

One of the characteristic features of this book is the large number of frozen sections which have been photographed and utilized in an admirable way to illustrate the principles of physical diagnosis. They are the work of Dr. George Fetterolf, Assistant Professor of Anatomy in the University of Pennsylvania, and show unusual skill in the art of anatomic preparation. About one-third of the book—the examination of the lungs and circulatory system—is written by Dr. Norris. The remaining two-thirds, on diseases, is the work of Dr. Landis. Dr. Krumbharr contributes a comprehensive chapter on the electrocardiograph in which he takes a conservative stand as to the usefulness of the information derived from this instrument. Throughout the work the authors have maintained the proper balance between the various factors entering into the diagnosis of disease, presenting the pathology, physical signs and other data, clearly and faithfully. The illustrations are superb, the paper and typography are attractive, and the result is a truly sumptuous volume doing credit to authors and publishers.

---

**The Practical Medicine Series.** Comprising ten volumes on the year's progress in medicine and surgery, under the general editorial charge of Charles L. Mix, A. M., M. D., Professor of Physical Diagnosis in the Northwestern University Medical School. Price of Series, $10.00. Chicago, The Year Book Publisher.

**Volume VII. Obstetrics.** Edited by Joseph B. DeLee, A. M., M. D., Professor of Obstetrics, Northwestern University Medical School, with the collaboration of Eugene Cary, B. S., M. D., Assitant Gynecologist, St. Luke's Hospital; Instructor in Gynecology, Northwestern University Medical School. Series 1917. Cloth, 12mo, 224 pages, illustrated, price $1.35.

**Volume VIII. Pharmacology and Therapeutics.** Edited by Bernard Fantus, M. S., M. D., Associate Professor of Medicine, Sub-department of Therapeutics, Rush Medical College, Chicago, Ill.

**Preventive Medicine.** Edited by Wm. A. Evans, M. S., M. D., LL.D., P. H. D., Professor of Preventive Medicine, Northwestern University Medical School. Series 1917. Cloth, 12mo, 384 pages, illustrated, price $1.50.

These books are intended primarily for the general practitioner, but the annual set including ten volumes allows the buyer to select those special subjects that he desires, if he does not wish them all.

Volume VII. gives a good choice of the representative papers of the past year. The value of these abstracts is greatly increased by the prudent comments of the editor, who does not believe all that he sees in print.

Volume VIII. covers about four times as many authors as the preceding book. It reflects the influence of our war, especially in the articles on "war time economy in drugs," "disinfection of ships," and "military hygiene."

